# Moving Beyond DNA Sequence to Improve Plant Stress Responses

**DOI:** 10.3389/fgene.2022.874648

**Published:** 2022-04-19

**Authors:** Faisal Saeed, Usman Khalid Chaudhry, Allah Bakhsh, Ali Raza, Yasir Saeed, Abhishek Bohra, Rajeev K. Varshney

**Affiliations:** ^1^ Department of Agricultural Genetic Engineering, Faculty of Agricultural Sciences and Technologies, Nigde Omer Halisdemir University, Nigde, Turkey; ^2^ Centre of Excellence in Molecular Biology, University of the Punjab, Lahore, Pakistan; ^3^ Key Laboratory of Ministry of Education for Genetics, Breeding and Multiple Utilization of Crops, Oil Crops Research Institute, Center of Legume Crop Genetics and Systems Biology/College of Agriculture, Fujian Agriculture and Forestry University (FAFU), Fuzhou, China; ^4^ Department of Plant Pathology, Faculty of Agriculture, University of Agriculture, Faisalabad, Pakistan; ^5^ State Agricultural Biotechnology Centre, Centre for Crop and Food Innovation, Murdoch University, Murdoch, WA, Australia; ^6^ Center of Excellence in Genomics & Systems Biology, International Crops Research Institute for the Semi-Arid Tropics (ICRISAT), Hyderabad, India

**Keywords:** biotechnology, epigenetics, food security, abiotic stress, biotic stress, stress memory

## Abstract

Plants offer a habitat for a range of interactions to occur among different stress factors. Epigenetics has become the most promising functional genomics tool, with huge potential for improving plant adaptation to biotic and abiotic stresses. Advances in plant molecular biology have dramatically changed our understanding of the molecular mechanisms that control these interactions, and plant epigenetics has attracted great interest in this context. Accumulating literature substantiates the crucial role of epigenetics in the diversity of plant responses that can be harnessed to accelerate the progress of crop improvement. However, harnessing epigenetics to its full potential will require a thorough understanding of the epigenetic modifications and assessing the functional relevance of these variants. The modern technologies of profiling and engineering plants at genome-wide scale provide new horizons to elucidate how epigenetic modifications occur in plants in response to stress conditions. This review summarizes recent progress on understanding the epigenetic regulation of plant stress responses, methods to detect genome-wide epigenetic modifications, and disentangling their contributions to plant phenotypes from other sources of variations. Key epigenetic mechanisms underlying stress memory are highlighted. Linking plant response with the patterns of epigenetic variations would help devise breeding strategies for improving crop performance under stressed scenarios.

## 1 Introduction

Agriculture plays a vital role in feeding the rapidly growing world population. For fibre, fuel, and food, we usually depend on major crops such as cotton, maize, sugarcane, rice, barley, wheat, and soybean. An increase in world population day by day puts tremendous pressure on current food crop production systems (Chaudhry et al., 2021a; Junaid et al., 2021). Additionally, climate change results in several weather adversaries, and frequent disease and pest attacks threaten crop production worldwide (Raza et al., 2020). These stresses interfere with plant’s physiological, biochemical, molecular, and cellular mechanisms, ultimately reducing overall growth, and production (Raza et al., 2020; Raza et al., 2021). With time, for better growth and cultivation, human beings carried out an artificial selection of thousands of plants to get desired traits in plants (Herron et al., 2020). In recent years, new ways and tools have been discovered for the betterment of crops. For instance, affordable genetic systems for profiling of the plant genomes have led to the development of robust molecular diagnostics for rapid and precise selection of desirable crop plants (Gökçe and Chaudhry, 2020; Gökçe et al., 2021). In parallel, targeted genetic modification has been greatly benefitted by the availability of the whole genome sequence information in different crop species. For instance, genome editing techniques such as clustered regularly interspaced short palindromic repeats (CRISPR) technology using RNA-guided nucleases have facilitated the alteration of a variety of important plant phenotypes (Ma et al., 2016; Saeed et al., 2020; Dangol et al., 2021).

Feeding the growing world population would require harnessing the latest discoveries in plant epigenetics. A growing body of literature suggests that epigenetics contributes to many vital traits in different plant species. The term epigenetics refers to heritable changes in the phenotype, which are not due to a change in DNA sequence (Heard and Martienssen, 2014). In other words, epigenetics involves alterations in gene expressions that are stably transmitted from generation to generation. Plant epigenetics combines different research fields that help us understand how plants adjust their phenotypes other than modifying their DNA sequence under extreme stress conditions. The molecular processes encompassing epigenetics are DNA methylation and histone modification (Henikoff and Greally, 2016). The structure of chromatin is regulated by methylation of DNA and modification of histones, and these modifications remain crucial to the repression or activation of a gene (Ganai, 2020). The review article presents the recent advances in plant epigenetics, emphasizing plant stress response. The underlying mechanisms are discussed in the following sections.

## 2 Mechanism

Rollin Hotchkiss in 1948 identified DNA methylation. After 30 years, Holliday and Pugh proposed that DNA methylation is an essential epigenetic hallmark (Holliday and Pugh, 1975)**.** Methylation of DNA is among the key epigenetic mechanisms regulating various bioprocesses. In plants, DNA methylation is initiated by the RNA-directed DNA methylation (*RdDM*) pathway. In all sequence contexts, DNA methyltransferases DOMAINS REARRANGED METHYLTRANSFERASE 2 (*DRM2*) catalyzes the process of methylation (Zhang et al., 2018a). *RdDM* is further divided into canonical and non-canonical pathways. In the canonical pathway, RNA polymerase IV synthesizes single-stranded RNA (ssRNA) that are changed into double-stranded RNAs (dsRNAs) by involving RNA Dependent RNA polymerase 2 (*RDR2*). DICER LIKE 3 (*DCL3*) involves in the cutting off this dsRNA. This dsRNA is than converted into 24 bases of small interfering RNA (siRNAs) (Zhang et al., 2018a). The second part of this pathway depends on the transcription of non-coding RNAs (ncRNAs) by the involvement of Pol V (Liu et al., 2018). Canonical pathway initiated by Pol IV- dependent 24 nt siRNAs whereas non-canonical pathway initiated by pol II and small RNAs (sRNAs) are involved. These sRNAs are produced from dsRNAs. sRNAs consist of 21–24 nt but are cut by different DCL proteins. These sRNAs are involved in triggereing post transcriptional gene silencing (PTGS) (Martínez de Alba et al., 2013; Cuerda-Gil and Slotkin, 2016). Methylation of cytosine involves a change in cytosine to 5- methylcytosine (5-mC), and it takes place when a methyl group is transferred at 5′ positions of S- adenosyl methionine. Cytosine methylation, though dependent on plant species, ranges from 6 to 25% therefore, plants have high levels of methylcytosine (Steward et al., 2000). Methylation of DNA cytosine occurs in three sequence contexts in plants, i.e., CpG, CpHpG, and CpHpH (H known as for T, A, and C). After replication, DNA methylation of CpG and CpHpG can easily be copied because the methylation at the symmetrical CG and CHG sites can be maintained during DNA replication. The methylation at the non-symmetrical CHH sites is not maintained during replication and occurs *de novo* (Karlsson et al., 2011). Plants store this epigenetic memory at the vegetative phase under different stresses and transfer it to the next generation, which gets established during the development of germline cells. DNA is methylated at both the gene body and the promoter regions, and due to this methylation, it allows the gene to remain suppressed. Thus, lower methylation helps increase the expression of a gene (Finnegan et al., 1998). Several processes are involved in the epigenetic mechanism. These mechanisms are cytosine methylation, chromatin proteins, and post-translational modifications (Abdolhamid Angaji et al., 2010).

### 2.1 Active and Passive DNA Methylation and Role of Non-Coding RNAs

Different developmental, physiological, and stress stimuli are involved in regulating DNA methylation in plants. Histone and DNA methylation are inter-reliant procedures. In *Arabidopsis* mutant *met1*, CpG causes the loss of methylation of *H3K9* (Soppe et al., 2002; Tariq et al., 2003). But the loss of methylation of *H3K9* in kryptonite (KYP) did not affect the methylation of CpG site (Jasencakova et al., 2003). Consequently, it shows that methylation of CpHpG site is partly dependent on the activity of KYP due to loss of *H3K9* methylation (Jackson et al., 2002). The process of demethylation and methyltransferase both control the methylation of DNA. Demethylation follows two routes, i.e., the passive and the active. During the process of cell division, methylated DNA can vanish from the genome. If maintenance machinery present in dividing cells can be blocked. During the duplication of DNA inhibition of enzymatic activity, expression loss or elimination of DNA methyltransferase repair machinery leads toward extinction of 5-mC marks. This loss of 5-mC sites is known as passive DNA methylation (Feng et al., 2010).

Active DNA demethylation occurs by glycosylase activity by taking out the methylcytosines (Zhu et al., 2000). A single nucleotide gap is filled by demethylated cytosine with the help of the base excision repairing process (Agius et al., 2006). Many RNA molecules in the eukaryotic genome do not participate in protein production and are called non-coding RNAs (ncRNA) (Mishra and Bohra, 2018). On a size basis, these ncRNAs are divided into two types. i.e., small ncRNA and long non-coding RNA (lncRNA). These groups were divided based on the size of the transcripts. Small ncRNAs possess less than 200 nucleotides (Bohra et al., 2021), whereas lncRNAs contain more than 200 nucleotides (Ghildiyal and Zamore, 2009; Quinn and Chang, 2016). Small and lncRNAs are important epigenetic players in regulating the plant stress response, growth, and development (El-Shami et al., 2007; Heo et al., 2013). Research has shown that lncRNAs serve as epigenetic regulators of gene expression at different stages (Karlik et al., 2019). LncRNAs work as *cis*-acting elements near the RNA synthesis sites (Zhao et al., 2020b). Further, *trans*-acting factors, they can also work away from synthesis sites (Suksamran et al., 2020). LncRNAs transcribed by polymerase II, III, IV, and V. They are further divided into five categories depending on their positions in genome near or away from protein-coding genes. The five categories are sense, antisense, bidirectional, intronic, and large intergenic lncRNA. Different lncRNAs are differentially expressed under various stresses and were suggested to play an important role (Urquiaga et al., 2021). These ncRNAs are involved in different epigenetic regulation mechanisms such as histone modification and DNA methylation (Ariel et al., 2014). Double-stranded RNAs synthesized by RNA Dependent RNA polymerases (RDRs) during this process, small interfering RNA (siRNA) arises (Song et al., 2019). siRNA are produced during transcription or transpositional reactivation of transposable elements (TE) during stress conditions (Hou et al., 2019b).

### 2.2 Histone Modification

Nucleosome architectures are altered in response to epigenetic changes; however, this alteration does not involve the DNA sequence (Zhao et al., 2021). Gene expression lowers or increases due to histone modification or DNA methylation (Singh and Prasad, 2021). During developmental processes or under any stresses, epigenetic changes in chromatin structure are regular and extremely active (Bhadouriya et al., 2021). Acetylation/deacetylation, SUMOylation, ubiquitin of histone proteins, phosphorylation/dephosphorylation are key processes involved in histone modification. These histone proteins are also altered chemically; they change their different physical or chemical properties (Boulanger et al., 2021). In the regulation of gene expression, the histones also release their subunits from the octamer core. These known modifications help increase DNA accessibility and speed up the selection process of binding proteins, which participate in DNA replication, transcription, or DNA repair (Pang et al., 2020). In the DNA methylation process, which occurs in the eukaryotic genome, at 50 positions of nitrogenous cytosine base, a methyl group attaches (-CH3) and forms a 5-mC (Pandey et al., 2017). These methylation processes can be asymmetrical and symmetrical; commonly CHH methylation process is known as asymmetrical, and on the other side, CHG and CG represent symmetrical methylation (Parent et al., 2021). As we discussed earlier *RdDM* is also common in plants (Singroha and Sharma, 2019). Cytosine methylation regulates gene expression by controlling the interaction of nucleic acid with transcription factors and chromatin proteins (Casati and Gomez, 2021). Patterns of DNA methylation are constant and particular to the exact cell type. These patterns are heritable and remain the same throughout life (Singh and Prasad, 2021).

### 2.3 Epigenetic Memory in Plants

**FIGURE 1:**
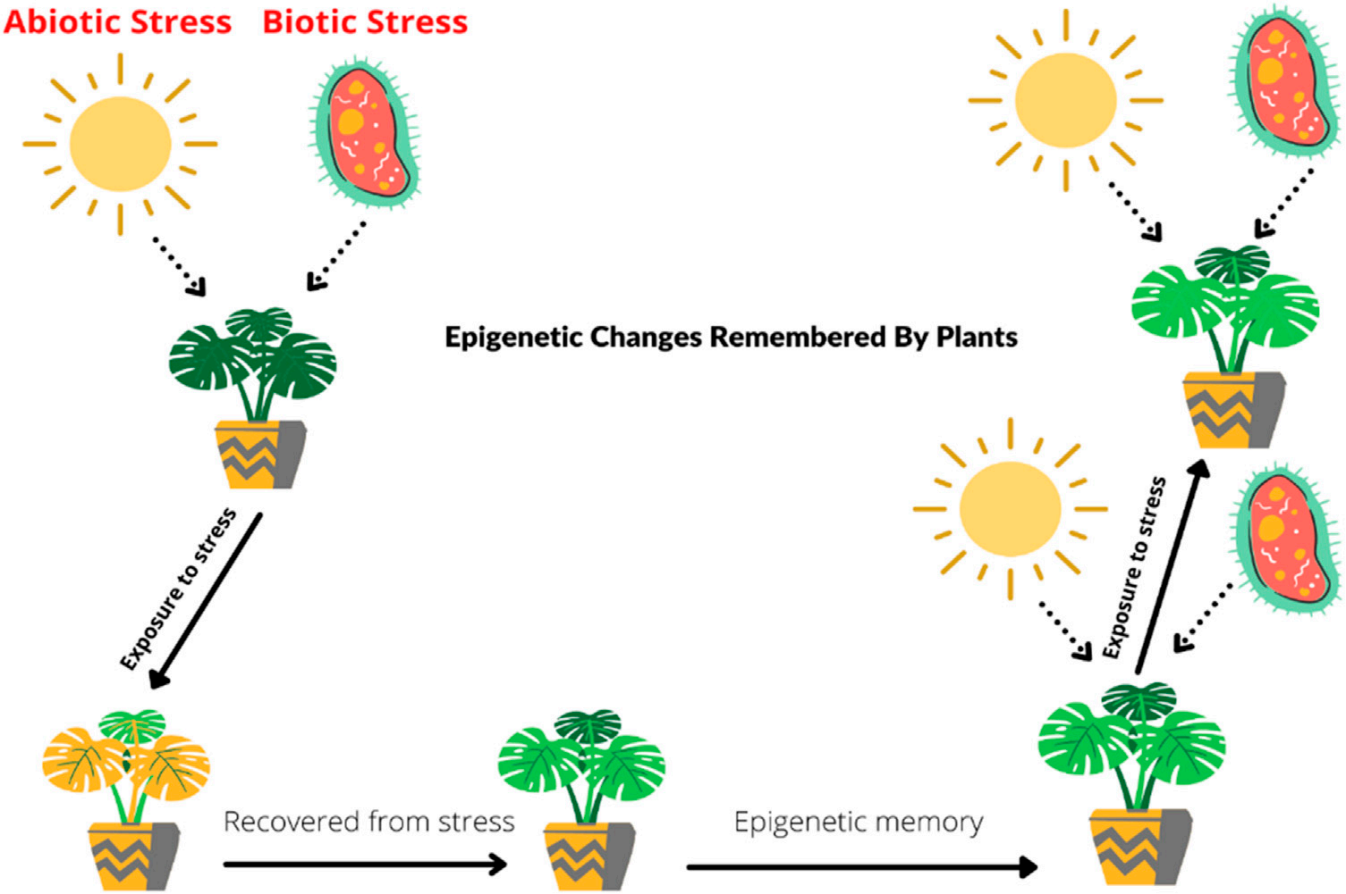
Mechanisms underlying epigenetic memory in plants during stress. Plants’ epigenetic memory helps protect them from different stresses. Whenever a plant faces stress regardless of its biotic or abiotic nature, it starts recovery against stress, and the plant epigenetic stress memory stores that information. Due to this stored memory, stress does not affect the plant on subsequent exposures.

Plant memorizes the epigenetic changes, and it helps them to adapt under biotic and abiotic stresses (Kinoshita and Seki, 2014; Crisp et al., 2016; He and Li, 2018). For instance, in *Arabidopsis thaliana*, two important factors that memorize during stresses are modification of histone and *HSFA2* (Heat shock factors). When a plant faces heat shock, the level of *H3K4* (H3 lysine K4 methylation) methylation remains high at least for 2 days. This process is also linked with transcriptional memory. The expression of heat stress response and transcriptional heat shock memory is dependent on the accumulation of *H3K4* methylation and *HSFA2*. *REF6*, known as RELATIVE OF EARLY FLOWERING 6, exhibits a positive response and transfers as long-term memory of epigenetic changes in *A. thaliana* (Liu et al., 2019a). *LSD-1* (Lysine-specific histone demethylase-1) in wheat recorded upregulation during heat stress as compared to normal plants. It is linked with modification of histone in the generation of transgenerational thermotolerance by heat priming. These changes induced by heat shock, transgenerational epigenetic memory, or changes in phenotype can be carried out at least two to three generations (Suter and Widmer, 2013; Zhonga et al., 2013). Priming of organismal stress response explains the events by which transient stimulus alters plant for future exposure to stress (Conrath et al., 2015). The term priming basically referred to immunity against pathogens but was later applied to abiotic stress. Priming is a reversible event because it only changed the phenotypical appearance of plant and does not change genetic makeup (Hilker et al., 2016). There are still many questions related to epigenetic memory. The specificity of stability of DNA and choramtin and their existence during mitosis and upkeep of memory. The mechanisms directly linked to chromatin changes which is further linked to transcriptional responses when plant faces stress are still not clear (Asensi-Fabado et al., 2017). A plant that once faces any harmful or stress conditions can recover from that stress, and epigenetic memory helps its future survival under stress conditions. As illustrated in Figure 1, the plant activates the epigenetic stress memory against future stresses, and the plant will remain protected.

## 3 Dynamics During Biotic Stresses

On exposure to biotic stress, the defense machinery evokes the immune system, such as basal defense machinery and pathogen-associated molecular patterns (Muthamilarasan and Prasad, 2013). DNA methylation changes in plants as a defense response against biotic stress. The role of DNA methylation has been reported in *A. thaliana* (Dowen et al., 2012). In *A. thaliana*, the *met1* and *ddc* mutants could not produce infectious symptoms by *Pseudomonas syringae* pv. *tomato* DC3000 due to the elimination of methylation capability of cytosine. Another gene named *ELP2* initiates DNA methylation in *Arabidopsis,* and pathogen-altered methylation of DNA takes place (Dowen et al., 2012; Yu et al., 2013). Akimoto and colleagues artificially reduced the DNA methylation at the promoter region of *R* gene (*Xa 21G*) of rice (Akimoto et al., 2007). During pathogen infection, the influence on the expression of defense-related genes was shown, caused by hypomethylation of DNA. When plants are infected with *Xanthomonas oryzae pv. oryzae*, rice mutant lines with constitutive silencing of *Xa21G* gene showed more resistance as compared to wild type (Akimoto et al., 2007). With advancements in molecular biology, scientists found new pathways and events in virus-infected plants. Plants generally employ siRNA-mediated DNA methylation against viruses or biotic stresses; in this mechanism, the plant methylated its genomic parts (Emran et al., 2012).

### 3.1 Biotic Stress-Related to the Epigenetic Configuration

Elucidation of the epigenetic alterations under biotic stress helps understand the plant-pathogen interaction (Zogli and Libault, 2017). In the analysis of plant-microbe relation, harmful *pst* introduced in Arabidopsis and DNA methylation changes occurred in all sequences of context. On the other side, when a non-harmful strain (i.e., bacterial strain) was introduced, only changes in CHG and CG methylation were detected (Dowen et al., 2012). Changes in methylation were common proximal to genes related to defense, and their activation correlated with transcription, so they have a role in reaction to pathogens (Yu et al., 2013).

Limited information is available on plant epigenetic effects created by fungal pathogens or oomycetes (Crespo-Salvador et al., 2018). *Arabidopsis* roots infected by cyst showed a huge change in DNA methylation and small RNA. In general, at the stage of infection, dynamic shifts take place (Joseph et al., 2021). However, DNA methylation change can be associated with several regions; transcriptional and epigenetic changes are related to each other and affect genes responsible for defense (Hewezi et al., 2017). In non-model crop species, understanding of epigenetic modifications is often limited, including patterns of DNA methylation (Herrera et al., 2016). In *Brassica rapa*, changes in DNA methylation were related to differences in floral morphology and less attraction of pollinators (Kellenberger et al., 2016). Detailed knowledge and high-resolution analysis will facilitate understanding plant-microbe interaction and underlying epigenetics changes (Richards et al., 2017). Plant-to-plant interactions facilitated by microbiota related to roots or allelochemicals obtained from plants may have some effect on chromatin arrangement (Venturelli et al., 2015).

### 3.2 Plant Epigenetic Influences on Biotic

Plants can be affected by neighbouring plants, microbes, and herbivores. Due to this, epigenetic changes affect plant phenotypes and plant’s interactions with other ogranisms (Latzel et al., 2012). Advances in plant epigenetics have deepened our understanding of the plant response against biotic stresses (Marfil et al., 2009). *AGO4* mutants of *A. thaliana* lacking methylation are susceptible to *pst* (Dowen et al., 2012). Similarly, overexpression of histone lysine demethylase enhanced resistance against blight in rice (Li et al., 2013). Relation between epigenetics and plant interaction with fungal pathogens is evident from enhanced susceptibility *A. thaliana* mutants (*dml1 dml2 ros1*) (Le et al., 2014). Post-translation histone modifications are also included in defense against pathogens (Xia et al., 2013). Evidence of inclusion of epialleles in biotic relations was discovered from mutants of *Arabidopsis* (Johannes et al., 2009; Reinders et al., 2009). Furthermore, these lines were also used to identify epigenetic QTLs that established a connection between epigenetic modifications and phenotypic variability (Latzel et al., 2012).

## 4 Plant Epigenetic Changes and Regulation During Abiotic Stresses

Climate change is crucial concerning, the adaptation of the crops to the changing climate scenarios as well as the growth of future crops to ensure food security. Environmental changes trigger drought, salt, heat, and cold stresses (Raza, 2020; Raza et al., 2020). Any fluctuation in temperature negatively affects plants growth and development, resulting in poor yield (Raza, 2021; Raza et al., 2021). Over the past few decades, several studies have explained the mechanisms of abiotic stresses, but reports on epigenetic regulation are still limited (Raza et al., 2021). Cold/chilling stress influences plant metabolic enzyme activities, responsible for gene expression (Raza et al., 2020; He et al., 2021; Raza et al., 2022). Cold stress is another important abiotic stress that retards plant growth and yield. To mitigate the risks associated with cold stress, plants have evolved signaling system that stimulates the expression of cold-stress-related genes. Plant response to cold stress is well-characterized, and research highlights the profound role of C- REPEAT BINDING FACTOR (*CBF*)-COLD RESPONSIVE (COR) pathways. Cold stress is reported to stimulate transcription factors (*TFs*) expression, which includes *CBF* family proteins. The *TFs* bind to the promoter region of downstream *COR* genes that activate its gene expression (Zhu, 2016). A recent study in *A. thaliana* described that chromatin remodeler PICKLE (*PKL*) is involved in *CBF*-dependent cold stress response (Yang et al., 2019). Histone methylation and histone modifications play a significant role against cold stress. For example, acetylation of histone is found enriched in several cold-responsive genes (Park et al., 2018). It is regulated dynamically by histone acetyltransferases (*HATs*) and histone deacetylases (*HDACs*) (Li et al., 2021). In recent research, *A. thaliana* plants were exposed to cold stress, and overexpression of histone deacetylase 2D (*HD2D*) exhibited lower lipid peroxidation with decreased accumulation of malondialdehyde contents that eliminated the oxidative burst (Han et al., 2016). Furthermore, plants under the influence of cold stress showed induction of histone acetylation in the promoter region of *COR47* and *COR15A* (Pavangadkar et al., 2010). The *H3K4me3* and *H3K27me3* have been reported to be involved in response to vernalization. The study elucidated their regional regulation and contributions to epigenetic memory. The vernalized *Brachypodium distachyon* induced epigenetic changes that regulate multiple genes to coordinate biological processes (Huan et al., 2018).

A global rise in temperature has attracted the attention of plant scientists to improve crop’s adaptation to future scenarios (Jha et al., 2014; Haider et al., 2022). In response to heat stress, Heat Shock Transcription Factor A1s (*HSFA1s*) are the main *TFs* controlled by phosphorylation or dephosphorylation and protein-protein interactions. A temperature higher than required for the normal growth functioning of a plant instantly disrupts its photosynthetic machinery with the absorption of increased light. It damages the photosystem II, thylakoid protein phosphorylation. Heat stress stimulates hyper-phosphorylation likewise activates heat shock *TFs*. Acetylation is the epigenetic modification that alters the *H2A* and *H3* histones, two important players associated with heat stress response in plants. For instance, actin-related protein 6 (*ARP6*) in *Arabidopsis* is reported to regulate gene expression. It encodes the *SWR1* complex that is necessary for the insertion of *H2A.Z* histone in nucleosomes as a replacement for *H2A* histone (Nie and Wang, 2021). It is also reported as an indispensable event for temperature sensing. Moreover, acetylation of *H3K56* is related to accumulating RNA polymerase II and activates *TFs* with exposure to heat stress (Haider et al., 2021). Research has demonstrated that the *RdDM* pathway and histone dynamics are involved in the responses against heat stress (Lämke et al., 2016; Yang et al., 2018). The heat shock proteins (*HSPs*) are primarily involved in conferring tolerance against heat stress in plants, regulating folding and unfolding of proteins (Singh et al., 2016). The heat stress stimulates the persistent expression of *H3K4me3* and *H3K9Ac* on *HSP18*, *APX2*, and *HSP70* genes (Lämke et al., 2016). In *A. thaliana*, the member of the F-Box family protein such as suppressor of *DRM1 DRM2 CMT3* (*SDC*) proteins facilitates the degradation of the protein. Heat stress for a prolonged period induces transcriptional expression of a subgroup of genes, which ultimately assist the plant in recovering from heat stress (Popova et al., 2013; Sanchez and Paszkowski, 2014). The *SDC* gene targets the *RdDM* pathway that can be silenced epigenetically under normal growth conditions. However, its activation under heat stress suggests a transcriptional response, which overcomes the silencing effect of *RdDM* at some loci.

Like heat, salt stress is another key challenge to global agriculture (Jha et al., 2014). The plant faces salt stress with the higher accumulation of salt contents, mainly increased sodium ions (Na^+^) content that cause ionic toxicity. The growth and development of plants are impaired following secondary oxidative stress (Chaudhry et al., 2021b; Hafeez et al., 2021). The participation of Histone acetyltransferase (*HAT*) in regulating salt tolerance has been elucidated in *A. thaliana* (Zheng et al., 2019). Higher uptake and accumulation of Na^+^ in the GENERAL CONTROL NONDEREPRESSIBLE 5 (*GCN5*) mutant as compared to wild-type plants impaired the growth of the mutant in response to salt stress. Additionally, *GCN5* can bind with cell wall synthesis genes such as CHITINASE-LIKE 1 (*CTL1*), and *MYB54*. Notably, lower *H3K9ac* and *H3K14ac* concentrations in mutant due to salt stress suggested that the *GCN5* is a conserved epigenetic regulator (Zheng et al., 2019). A recent study on *GCN5* in wheat has shown that target genes responsible for producing ROS species such as H_2_O_2_ (Zheng et al., 2021). The calcium ions (Ca^2+^) CALCINEURIN B-LIKE PROTEIN (*CBL*) CBL INTERACTING PROTEIN KINASE (*CIPK*) component performs an essential role in regulating cellular ionic homeostasis (Zhu, 2016). Higher Na^+^, lower K^+^, excessiveness of Mg^2+^, and higher pH levels stimulate cytosolic Ca^2+^ signaling for the activation of SALT OVERLY SENSITIVE3 (*SOS3*)-*SOS2*, *CBL2/3*-*CIPK3/9/23/26*, *CBL1/9*-*CIPK23*, and *SCaBP1*-*CIPK11/14* that causes phosphorylation and regulation of the activity of H^+^ ATPase, Mg^2+^ transporter, *Arabidopsis* K^+^ TRANSPORTER (*AKT1*, K^+^ channel), and *SOS1* (Na^+^/H^+^ antiporter) (Zhu, 2016). HIGH-AFFINITY K^+^ CHANNEL 1 (*HKT1*), which facilitates Na^+^ influx in plants, is vital transporter for coordinating with the *SOS* pathway to confer salt tolerance (Rus et al., 2001). In *A. thaliana* wild-type plants, a small RNA target region was identified at approximately 2.6 kb upstream of *HKT1* that was reported to be highly methylated (Baek et al., 2011). It was reported that a lower DNA methylation level in *RdDM* mutant *rdr2* led to an enhanced expression of *HKT1*, thus highlighting the role of *RdDM*-mediated regulation of gene expression (Miryeganeh, 2021). Another study in wheat revealed that salt stress-induced cytosine methylation caused suppression of *TaHKT2* expression in roots and shoots of both tolerant and sensitive genotypes (Kumar et al., 2017). The *TFs* induced by salt stress include *MYB74* of the *R2R3-MYB* family. The *MYB74* promoter is extremely methylated due to the *RdDM* pathway, and in salt stress, 24-nt siRNA levels and DNA methylation were almost imperceptible at *MYB74* is accompanied by the upregulated expression of *MYB74* (Xu et al., 2015).

Histone dynamics have been associated with drought stress response in plants (To and Kim, 2014). ABA-mediated signaling, playing an important role in drought stress in plants, is influenced by epigenetic modulation caused by either DNA methylation or histone acetylation. For example, analysis of *ABA*-deficient mutant maize (vp10) revealed differential methylation of several stress-responsive genes and *TE*. The key enzyme involved in the synthesis of *ABA* is NINE CIS-EPOXYCAROTENOID DIOXYGENASE 3 (*NCED3*) (Nambara and Marion-poll, 2003). Plant acclimatization to drought stress improved following deposition of *H3K4me* in the *NCED3* gene that caused higher gene expression (Ding et al., 2011). Moreover, the elevated expression level has been noted in the genes *RAP2.4*, *RD29A*, *RD29B*, and *RD22* in response to drought stress (Takahashi et al., 2000). The increased levels of *H3K4me3* and *H3K9Ac* in the promoter regions of *RAP2.4*, *RD22*, *RD29A*, and *RD29B* also contributed to the activation of genes expression. It was suggested that histone marks in response to drought stress also varied with the intensity of stress. As *H3K4me3* and *H3K9Ac* levels were higher with the exposure to severe drought in contrast to mild stress conditions (Kim et al., 2012). In *A. thaliana*, lower deposition of *H3K27me3* in the gene body region of drought-associated *TFs* resulted in resistance to drought (Sebastian Ramirez-prado et al., 2019). The *H3K27me3* reader protein is LIKE HETEROCHROMATIN PROTEIN 1 in the *PRC1* complex (Mozgova and Hennig, 2015). Moreover, drought-stressed plants had modified DNA methylation levels that ultimately altered expression levels of several drought-responsive genes (Liang et al., 2014). The miniature inverted-repeat transposable element (*MITE*) inserts in the promoter region of the *NAC* gene suppress the expression with the deposition of *RdDM* and *H3K9me3* (Mao et al., 2015). Likewise, zinc finger gene *ZmMYB087* is related to the metabolism group *MYB* transcription factor to regulate the biosynthesis of the secondary cell wall. CW-type zinc finger protein participates in the methylation of histone *H3* that is essential for epigenetic memory (Sallam and Moussa, 2021).

Optimal nutrient supply is essential for plant growth and development, but the excessive nutrients in the soil impair it by causing nutrient stress (Salim and Raza, 2020). Higher nitrogen in the soil down-regulated the expression of root nitrogen transporter, *NRT2.1*. The gene repression necessitates the involvement of the HIGH NITROGEN INSENSITIVE 9 (*HNI9*) in depositing *H3K27me3* on the *NRT2.1* gene (Widiez et al., 2011). Iron homeostasis in *Arabidopsis* was negatively regulated by *PRMT5*-mediated *H4R3* symmetric dimethylation (*H4R3sme2*) (Fan et al., 2013). The *PRMT5* linked with the *bHLH* genes, i.e., *AtbHLH38* and *AtbHLH100*, for the symmetrical demethylation of *H4R3* with no change in its gene expression (*PRMT5*) (Fan et al., 2013). Histone acetyltransferase *GCN5* is involved in regulating iron homeostasis by FERRIC REDUCTASE DEFECTIVE 3 (*FRD3*) (Xing et al., 2015). The *GCN5* can directly bind to the promoter region of iron-associated genes, which includes *FRD3*, to modulate the acetylation levels of *H3K6* and *H3K14* (Xing et al., 2015). The *H3K4me3* acetylation and histone variant *H2A.Z* have a key role in response to phosphorus-deficient soil conditions. The protein PHD ALFIN-LIKE 6 (*AL6*) binds to the *H3K4me3* mark that influences the maturation of transcript and stability of vital genes necessary for elongation of root hairs (Chandrika et al., 2013). Additionally, histone modifications in response to deficient phosphorus showed vast remodeling of DNA methylation (Secco et al., 2015). Gene expression levels of DNA methylase were induced on exposure to limiting phosphorus conditions (Yong-Villalobos et al., 2015). Table 1 enlists various crops where epigenetic mechanisms controlling response to various stresses have been elucidated.

**TABLE 1:** Stress-related epigenetic mechanisms for improved crop development under stress conditions.

Crop	Mechanism	Reference
Drought stress		
Rice	DNA methylation at a specific site	Wang et al. (2011)
Barley	Excessive accumulation of *H3* and loss of *H3K9me2*	Temel et al. (2017)
Maize	Enrichment of *H3K4me3* and *H3K36me3*	Xu et al. (2017)
Maize	Modified dynamics of *H3K4me3* and *H3K9ac*	Forestan et al. (2018)
Soybean	Upregulated isomiRNAs	Sosa-Valencia et al. (2017)
Pea	Cytosine hypermethylation	Labra et al. (2002)
Cotton	Histone modification	Chen et al. (2019)
Salt stress		
Wheat	Increased cytosine methylation of *HKT* genes	Kumar et al. (2017)
Rice	Differentially methylated regions of DNA	Ferreira et al. (2019)
Rice	Demethylation in the promoter of *OsMYB91* gene and modification of histone	Zhu et al. (2015)
Temperature stress		
Soybean	Cytosine hypomethylation	Hossain et al. (2017)
Wheat	Higher histone demethylation of several genes	Wang et al. (2016)
Maize	Modification of *H3K4me2* and *H3K9ac*	Hou et al. (2019a)
Maize	Higher acetylation of histone and reduction of *H3K9me3*	Wang et al. (2014)
Maize	Decreased acetylation of histone	Hu et al. (2011)
Maize	Higher accumulation of *H3K9ac*	Hu et al. (2012)
Mustard	Non-coding RNA mediated regulation	Bhatia et al. (2020)
Rice	Methylation of promoter region	Guo et al. (2019)
Biotic stress		
Potato	BABA primed histone modification against *Phythophtra infestans*	Meller et al. (2018)
Tomato	Methylation in cytosine residue to improve resistance against Tomato spotted wilt virus	Werghi et al. (2021)
Olive	Methylation to improve resistance against *Verticilium dahliae*	Crespo-Salvador et al. (2020)
Tomato	Improved resistance against pathogen creating mutants of Histone domain	Bvindi et al. (2022)

## 5 Developing Experimental Populations to Disentangle Epigenetic Effects From DNA Sequence Variation

Development of experimental populations based on the two genotypes that have little DNA sequence polymorphism but show extensive variation in their methylation patterns could help greatly to overcome the confounding effect of DNA methylation and DNA sequence variation, exemplified by epigenetic recombinant inbred lines (epiRILs) in *Arabidopsis* based on Columbia (with wild type DDM1 allele) and Col-ddm1 mutant (Johannes et al., 2009). Similarly, *Arabidopsis* epiRILs showed distinct phenotypic variations with altered resistance stress (Lloyd and Lister, 2022). Such experimental populations laid a foundation to study epigenetic contributions to novel phenotypic variations. The combined studies of epiRILs and natural accessions suggested that epigenetic diversity is an important component of functional biodiversity. Additionally, comprehensive analysis of genes responsible for epigenetic machinery in epiRILs led to the mechanisms engaged in heritable DNA methylation and the resulting impact on plant phenotype (Johannes et al., 2009). DNA methylation is inherited in a stable Mendelian fashion in some genomic regions (Colomé-Tatche et al., 2012). The epiRIL population of *ddm1*, showed stable inheritance of differentially methylated regions for several generations without disrupting the DNA sequence and established a role for epigenetic quantitative trait loci (epiQTL) in phenotypic manifestation (Zhang et al., 2013).

The epiRILs of Arabidopsis differ for DNA methylation but show very minute changes in their DNA sequences. The development of epiRILs is similar to the creation of classic RILs, which includes the crossing of two genetically divergent parents and subsequently, inbred lines are established. DNA methylation exhibited developmental phenotypic changes that suggested the suppression of phenotypic plasticity, and it can be assessed with the construction of epiRILs (Bossdorf et al., 2010). The F2 progenies can be screened for the homozygosity of alleles to confirm the function of DNA methylation machinery and subsequently maintain the epigenetic chimeric chromosome developed by recombination at the F1 meiosis stage. The epiRILs harbor phenotypic variations for plant morphological characteristics, growth rate, and abiotic stress responses. Studies on epiRILs showed that epigenetic variations contributed to the functional diversity that had a similar impact on populations as noticed in genetic diversity. Similarly, higher epigenetic changes resulted in improved plant phenotypes translating to higher productivity and resilience in populations (Latzel et al., 2012). The heritable phenotypic variations among epiRILs in response to drought stress revealed phenotypic plasticity with the significant variation in root: shoot ratio that has the potential for developing stress-resilient plants (Zhang et al., 2013). The crop improvement is based on the stable transmission of epialleles in inheritance. With the introduction of methods for creating epiRILs in crop plants, the generation of epimutagenesis and engineered epigenetic changes would contribute to bridging the gaps for harnessing epigenetic variations for trait improvement to confer stress tolerance.

## 6 Different Methods of Detection of Epigenetic Changes in the Genome

In recent years, several methods have been discovered to profile large-scale epigenetic modifications. With rapid progress in the field of biological sciences now, these methods are accurate, precise and affordable. Sodium bisulphite seqeucning and methylated immunoprecipitation sequencing (MeDIP-seq) in combination with latest sequencing technologies like single-molecule real-time sequencing (SMRT-seq) and Nanopore sequencing (Liang et al., 2019) are used genome-wide methylation profiling.

### 6.1 Sodium Bisulfite Method

DNA methylation can be detected by sodium bisulfite, which converts demethylated cytosines into uracil (Kushwaha et al., 2016). However, it did not affect the 5-mC (Papanicolau-Sengos and Aldape, 2022). The sodium bisulfite method of detection of epigenetic changes is a standard technology for the detection of 5-mC due to its capacity to provide huge information about methylated DNA segments (Zhao et al., 2020a). Different methods after conversion are also used for further analysis, such as PCR analysis, sequencing of the methylated genome is widely used. It can provide information about even a single methylated nucleotide (Matzke and Mosher, 2014). Frommer and colleagues pioneered DNA methylation analysis based on bisulfite genomic sequencing. In this method, firstly, DNA is denatured by using alkali and treated with bisulfite. After that, in the second part, the region of interest was amplified by PCR using bisulfite-specific primers (Frommer et al., 1992). On the other sides, this sequencing method has several disadvantages, and we cannot differentiate between 5-mC and 5-hydroxymethyl cytosine. Unsuccessful reaction of bisulfite may cause a failure to covert entire demethylated cytosines to uracil, thus leading to false-positive results (Fraga and Esteller, 2002). When template DNA is treated with bisulfite, it is difficult to design primers for multiplex PCR reactions (Callinan and Feinberg, 2006). To overcome these problems, a modified method of bisulfite sequencing, reduced representation bisulfite sequencing (RRBS), was proposed (Meissner et al., 2005). This method gained traction among researchers due to its low cost and coverage of all regions. This method only required specific genomic sequences (Hahn et al., 2014). This can help detect different levels of methylation and can analyze the specific genes under stress and control conditions. RRBS technique provides useful data for further methylation measurements. Examination of wild tobacco plants under salt and low temperature analyzed by bisulfite sequencing revealed demethylation at GC sites in coding regions, and demethylation was found in promoter regions (Rehman and Tanti, 2020). In 2019, Liu and colleagues demonstrated two methods for the detection of 5-mC and 5-hydroxymethyl cytosine. One is ten-eleven translocation (TET)-assisted pyridine borane sequencing (TAPS) (Liu et al., 2019b) and its modified version long-read TET-assisted pyridine borane sequencing (lrTAPS) (Liu et al., 2020). With the help of pyridine borane, TAPS uses TET oxidation of 5-methyl cytosine and 5-hydroxymethyl cytosine to produce 5-carboxylcytosine that ultimately reduces to dihydrouracil (DHU). After that, the PCR reaction converts DHU to thymine and at last recognizes 5-mC and 5-hydroxymethyl cytosine (Liu et al., 2019b).

### 6.2 Methylation Detection Based on Antibody

Methylated DNA immunoprecipitation (MeDIP) is one of the sensitive and widely used methods for methylome studies of plants. It is a purification method of gDNA fragments that contain methylated sites (Zhang et al., 2006). In this method, the fluorescent dye uses chemical derivatives of normal and modified deoxyribonucleotides to covalently bind to 6-methylated adenine (6 mA). Further, this 6 mA was quantified by using Capillary Electrophoresis with Laser-induced fluorescence (CE-LIF). The quest for alternatives like light sources instead of expensive lasers would impart cost-effectiveness to this method (Nguyen and Kang, 2019).

### 6.3 Other Methods of Methylation Detection

Bisulfite sequencing can only detect changes at C, but it cannot be distinguished between 5mC and 5hmC, and also it needs a reference genome (Flusberg et al., 2010). There are several methods of methylation detection, such as HPLC-MS/MS that can detect even the low to the lowest amount of 6-methyl adenine in plant genome (Huang et al., 2015). Another method, dot blot assay, is used with specific antibodies applied, but this approach has a limit of detection (Liang et al., 2019). These two methods are widely used for the detection of 6-methyl adenine, but these approaches are unable to reveal the location of methylated sites.

### 6.4 Different Next-Generation Sequencing Tools

The nucleotide sequencing technologies have evolved rapidly over the past 20 years (Varshney et al., 2007; Bohra et al., 2020; Varshney et al., 2021). First-generation sequencing technologies invented by Sanger and Maxam Gilbert revolutionized the molecular biology field (Sanger et al., 1977). In the mid-90s first Sanger sequencing platform (ABI 370) was made commercially available (Watts and MacBeath, 2003). The Sanger sequencing is the preferred technique for plant molecular biologists. It deals with the DNA genome of plants to the sequence. The ABI 370 xl DNA sequencer has maximum accuracy of up to 99.99%. It can generate reads as small as 1.9 kb to as long as 84 kb, and in 3-h run, it can create up to 300–400 bp reads (Liu et al., 2014). The major drawback associated with this sequencing technology is its time and resource-intensive nature. These bottlencks were overcome by the introduction of the second-generation of sequencing or next generation seqeucning (NGS) in 2005. This NGS technologies not only cut down the cost of sequencing but also produce million of reads in less time (Kchouk et al., 2017). Further development of the third-generation sequencing methods overcame many problems related to the second-generation sequencing methods, including sample preparation, product amplification, and time. The third-generation sequencing technique SMRT-seq (Single-molecule real-time sequencing) offers exact sequences and measures the nucleotide energy rate during sequencing. This sequencing method detects DNA changes at a single base. The NGS-based methologies have helped greatly to study genome-wide methylation patterns, as exemplified in *Arabidopsis thaliana* (Liang et al., 2018), rice (Zhang et al., 2018b), and fig (*Ficus carcia*) (Usai et al., 2021). The recently developed PacBio sequencing method requires only 5 h from sample to produce reads, and also it reduces costs. The only disadvantage of this system is a higher rate of error (14%) (Chin et al., 2016; Kchouk et al., 2017). The *in situ* sequencing (ISS) represents a fourth-generation platform that directly sequences the nucleic acids with a higher accuracy (Mignardi and Nilsson, 2014; Ke et al., 2016).

## 7 Combining Epigenetics and Gene Editing to Improve Stress Response

**FIGURE 2:**
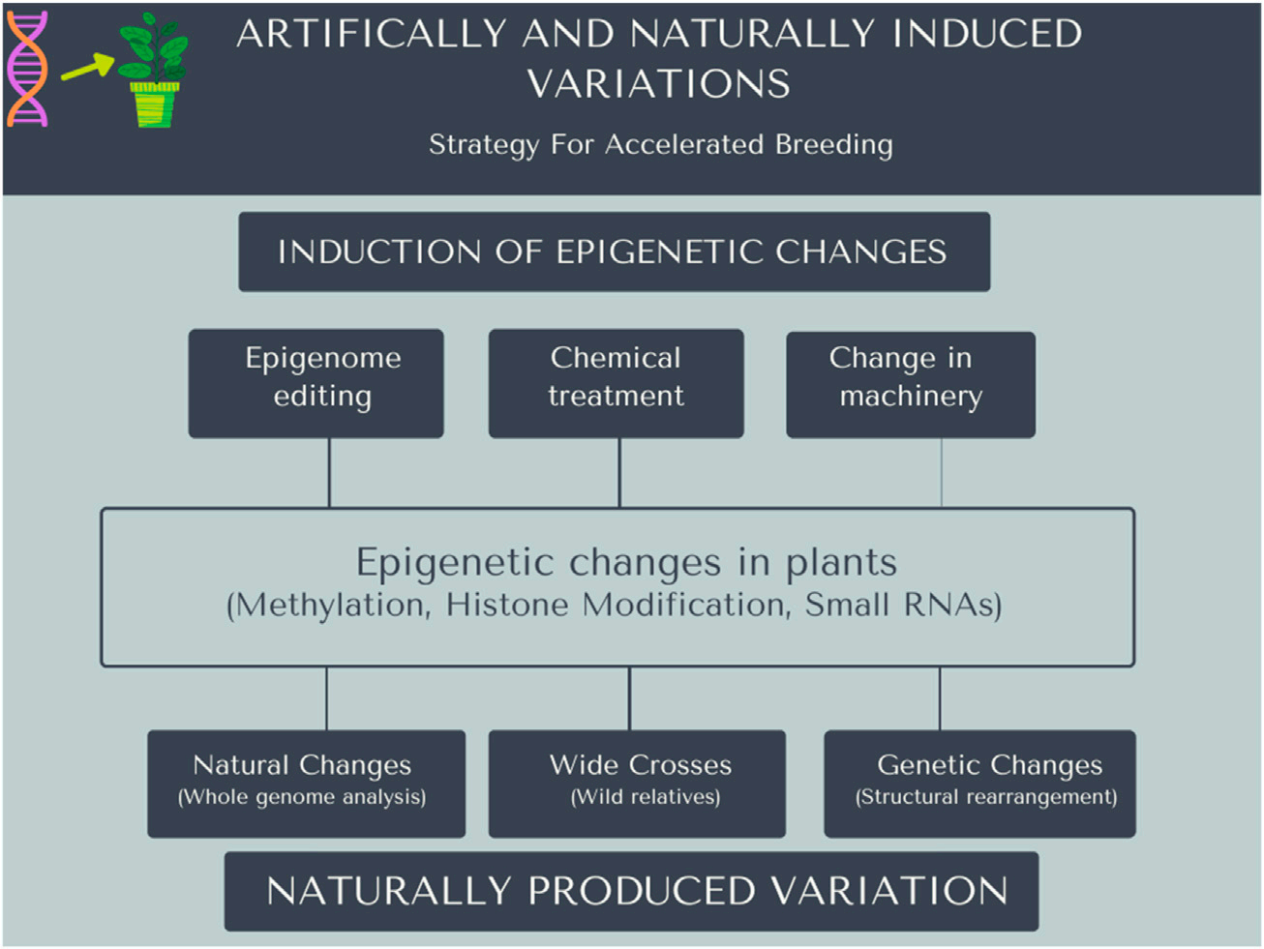
Artificially or naturally induced mutations in the plant genome can be helpful for accelerated breeding. Epigenetic changes can be induced in the plant genome using different methods, i.e., naturally or artificially. Artificially it can be induced using editing techniques, chemically treated plants to create mutations, and alter the machinery using different molecular approaches. Wide crosses of plants and naturally occurring changes in the genome in nature can be useful for accelerated breeding.

Biotic and abiotic stresses deteriorate the yield and quality of crop plants. Conventional breeding approaches are increasingly constrained to rapidly develop plant varieties having adaptation to changing climatic conditions. It is the need of the hour to utilize different approachesfor accelerated crop improvement (Watson et al., 2018). The development of stress-resilient crops requires plant breeding approaches to exploit variations beyond DNA sequence (Fiaz et al., 2019). Plant stress memoryrelated genes or factors can be used by genome editing techniques for a better understanding of the regulatory mechanisms underlying stress response. Plant response to various stresses (biotic and abiotic) is known to involve several genes (Raza et al., 2021). Epigenetic changes can be induced in plants, and these can be used as a helpful strategy for the improvement of crops and can accelerate the breeding process, as shown in Figure 2. Nevertheless, these epigenetic changes can be induced artificially by editing of epigenome or chemically treated to create mutation (Figure 2). The CRISPR/Cas9 protein is successfully utilized as a dCas9 to modify epigenetic changes. The dCas9 protein is attached with the epigenetic modifier to targeted modifications that result in altered gene expression (Adli, 2018).

After the modification in Cas9 protein and the emergence of CRISPR/dCas9 technology, scientists start regulatory and reporter genes to investigate the ability of dCas9 for epigenome editing (Zezulin and Musunuru, 2018). It was reported that the plant genome was modified by methylation and demethylation at target DNA and resultantly developed late-flowering phenotypes (Adli, 2018). It has been reported that the fusion of repressor domains (such as KRAB/SID) with dCas9 led a significant improvement of transcriptional repression (Gilbert et al., 2013). Furthermore, the fusion of active transcriptional domain *VP16/VP64* activates the expressional level of the gene of interest that permits the screening of stress-tolerant genotypes (Miglani et al., 2020). Nowadays, synthetic regulation of transcriptional modifications has been successfully considered for traits improvement by the CRISPR technique (Piatek et al., 2015). This technique has been used for precise and spatial modification by avoiding undesirable pleiotropic effects. For instance, the promoter of *OsRAV2* gene, a *TF* responsible for salt stress, was genetically manipulated by CRISPR/Cas technique. The impaired growth of mutant lines by salt stress confirmed the important role of *GT1* during normal plant growth and development (Duan et al., 2016). Conventional plant breeding has served humanity and this planet for a century and has always helped produce high-yield crops and fulfill the requirement of food for humans (Hickey et al., 2019). To this end, emerging plant breeding technologies such as epigenome editing can help to achieve food security targets by protecting plants from biotic or abiotic stresses.

## 8 Conclusion and Prospects

Understanding the interactions between epigenetics and plant stress response has huge potential to develop modern crops adapted to future climatic conditions. Unlike abiotic stress, epigenetic regulation of biotic stress tolerance of plants is more complex, and it remains more challenging to carry out experiments for different species *in vitro.* In the future, biotechnologists, ecologists, and molecular biologists may collaborate to find out the mechanism involved between epigenetics and stress response. Methylome profiling has remained a challenge owing to the cost and technological considerations. However, recent advances in high-throughput assays have helped relieve this bottleneck. Interdisciplinary research efforts may help to sequence the genomes of diverse accessions and also play a vital role in establishing genomic tools to find out the epigenetic changes to stresses (Schrey et al., 2013). The growing information on whole genomes and gene content would inspire future researchers to decipher the epigenetics-mediated response of plants to a variety of biotic stresses. The foremost objective will be to survey epigenetic variation and quantify its effects on phenotypes in different crops (Kawakatsu et al., 2016). Furthermore, the candidate loci for the target traits can be identified, and epigenetic profiling may be precisely targeted to the candidate regions (Aller et al., 2018). A key challenge to decipher the role of epigenetic regulation is the application of stress treatments in controlled conditions. In the natural environment, plants often deal with multiple stresses at one time. With the rapid advances in genome-wide methylation profiling and gene editing techniques, we envisage a better understanding of the epigenetic changes controlling plant stress response, which will be crucial to precisely manipulate the epigenetic regulatory mechanism for improved crop performance.
